# The use of mobile phone data to inform analysis of COVID-19 pandemic epidemiology

**DOI:** 10.1038/s41467-020-18190-5

**Published:** 2020-09-30

**Authors:** Kyra H. Grantz, Hannah R. Meredith, Derek A. T. Cummings, C. Jessica E. Metcalf, Bryan T. Grenfell, John R. Giles, Shruti Mehta, Sunil Solomon, Alain Labrique, Nishant Kishore, Caroline O. Buckee, Amy Wesolowski

**Affiliations:** 1 Department of Epidemiology, Johns Hopkins Bloomberg School of Public Health, Baltimore, MD USA; 2 Department of Biology and the Emerging Pathogens Institute, University of Florida, Gainesville, FL USA; 3 Department of Ecology and Evolutionary Biology and the Woodrow Wilson School of International and Public Affairs, Princeton University, Princeton, NJ USA; 4 Department of International Health, Johns Hopkins Bloomberg School of Public Health, Baltimore, MD USA; 5 Department of Epidemiology and the Center for Communicable Disease Dynamics, Harvard TH Chan School of Public Health, Boston, MA USA

**Keywords:** Data mining, Ecological epidemiology, Viral infection

## Abstract

The ongoing coronavirus disease 2019 (COVID-19) pandemic has heightened discussion of the use of mobile phone data in outbreak response. Mobile phone data have been proposed to monitor effectiveness of non-pharmaceutical interventions, to assess potential drivers of spatiotemporal spread, and to support contact tracing efforts. While these data may be an important part of COVID-19 response, their use must be considered alongside a careful understanding of the behaviors and populations they capture. Here, we review the different applications for mobile phone data in guiding and evaluating COVID-19 response, the relevance of these applications for infectious disease transmission and control, and potential sources and implications of selection bias in mobile phone data. We also discuss best practices and potential pitfalls for directly integrating the collection, analysis, and interpretation of these data into public health decision making.

## Abstract (web summary)

In this Perspective, the authors review the different applications for mobile phone data to support COVID-19 pandemic response, the relevance of these applications for infectious disease transmission and control, and potential sources and implications of selection bias in mobile phone data.

## Introduction

The coronavirus disease 2019 (COVID-19) pandemic caused by the novel coronavirus (SARS-CoV-2) has created an unprecedented challenge for governments, public health agencies, medical officials, and populations globally^1,2^. The public health response is seeking to effectively mitigate and contain the pandemic while balancing social and economic costs^3–5^. Control strategies thus far have primarily consisted of non-pharmaceutical interventions (NPIs), which have slowed down the epidemic in many settings. Most NPIs rely on reducing contact between infected and susceptible individuals through mass social distancing, including restrictions on social gatherings, closures of schools and businesses, shelter-in-place or stay-at-home orders or lockdowns, travel restrictions, active monitoring, and increased testing, contact tracing, and isolation measures^6–9^. These interventions are effective when they result in large-scale human behavioral changes that reduce the close contacts and mobility patterns that facilitate disease transmission, but are challenging to maintain^10^. Quantifying these patterns to assess NPI effectiveness, particularly on the spatial, temporal, and population scales necessary to fully inform public health response, is an important challenge for this pandemic response.

As a result of the rapid spread and grievous toll exacted by the COVID-19 pandemic, there has been increasing interest in developing innovative methods and tools to inform public health response through digital data, including mobile phone data both passively collected by mobile phone operators and actively collected via recently developed applications^11^. Mobile phone data remain one of the best sources of information on large-scale population behaviors^12^. These data can be collected in high- and low-income settings and can capture, in near real-time, changes in mobility and clustering patterns for large swaths of the population. We and others have previously used aggregated and anonymized geolocation information from passively collected mobile phone data to successfully inform and model the spatial and temporal dynamics of endemic and emerging infectious diseases, including malaria^13–16^, cholera^17^, measles^18–23^, dengue^24,25^, and Ebola^26,27^. Through these prior applications, an understanding of privacy-conscious ways to utilize these data and inform public health policy while forming productive collaborations with operators, public health officials, and academic partners has been developed.

**Fig. 1:**
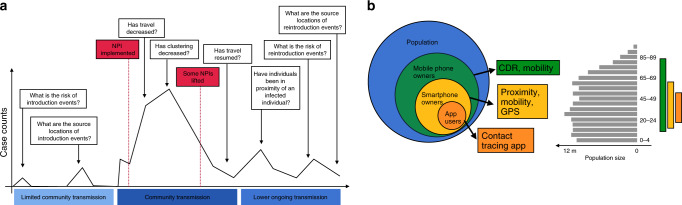
The uses of mobile phone data to inform COVID-19 public health response and their possible biases. **a** Over the course of the epidemic, mobile phone data and applications may be relevant to help answer a number of important epidemiological questions needed to guide the implementation and evaluation of various interventions. **b** However, these data should be considered in light of ownership and use biases that may or may not limit generalizability to the overall population. Mobile phone owners and users only represent a subset of the population and may have additional age (shown here for a synthetic population for illustrative purposes), socio-demographic, or geographic biases. Applications that require the use of a smartphone or application may further limit the generalizability of these data since they represent smaller subsets of the user population.

Mobility analysis, quantifying clustering of social contacts, symptom tracking, surveying, and contact tracing applications have all been proposed and employed to some degree to inform the response to COVID-19 (see Fig. 1a). These applications, metrics developed to analyze these data, and proposed best practices have recently been reviewed by an interdisciplinary team of experts^28^. To build on this work, we examine the applicability of mobile phone data for public health response by reviewing the common applications of mobile phone data relevant to outbreak response; the kinds of behaviors captured within these data and proposed applications; the validity of these data for public health response and epidemiologic research, including sources and implications of selection bias; and potential concerns and best practices for direct integration of these data with public health response.

## Utilizing mobile phone data to inform COVID-19 response

Mobile phone data can be used to inform different aspects of COVID-19 response (Table 1). At the population level, quantifying changes in human mobility or clustering can help evaluate the impact of an NPI and identify hotspots where additional or different interventions may need to be applied. At the individual level, mobile phone data may be used to understand patterns of individual contacts and enhance contact tracing.

**Table 1:** Summary of types, metrics, and proposed applications of mobile phone data.

Data type/information	Metrics	Applications	Advantages	Limitations
**Call data records (CDR):**				
• Collected routinely by mobile phone operators • Consists of a time stamp, GPS location of local cell tower, and unique identifier for all subscribers	• Origin-Destination Matrix • Radius of Gyration • Subscriber Density	• Assess changes to population-level mobility and clustering behaviors • Understand risk of importation from different regions • Retrace likely introduction and spread of an outbreak in new areas • Inform projections of disease risk or burden across space	• Typically readily available • High coverage to estimate large, population-level mobility patterns for entire countries or region • Available frameworks provide aggregated, anonymized metrics	• Assumes aggregate mobility behaviors represent that of infected/potentially infectious individuals • Cannot distinguish high vs low risk of transmission • Limited data in Internet-enabled or low cell-tower-density areas • Limited use in understanding transmission chains • Selection bias for whom data is available (mobile phone user)
**GPS location data:**				
• Collected passively through some smartphone applications • Consists of time stamp, GPS location of phone, and unique identifiers for all application users	• Origin-Destination Matrix • Radius of Gyration • User Density, Proximity	• Assess changes to population-level mobility and clustering behaviors • Understand risk of importation from different regions • Retrace likely disease introduction and spread in new areas • Inform projections of disease risk or burden across space	• Provides higher resolution spatial data than CDRs • Provides population-level insight into the average clustering and movement of individuals	• Selection bias in the population for whom data is available (smartphone users who opted into app) • Fewer standardized frameworks for managing privacy and anonymization of potentially sensitive information
**Bluetooth data:**				
• Collected passively by Bluetooth-enabled phones • Consists of the time stamp, distance, and duration of interaction between two devices with unique identifiers	• User Density, Proximity • Proximity Network Characteristics (degree, clustering)	• Assess changes to population-level clustering behaviors due to NPIs • Assess changes to pairwise contact rates in a given population over time	• Large-scale collection of data on pairwise interactions and clustering • Interactions potentially more relevant to disease transmission	• Selection bias in the population for whom data is available (mobile phone user, Bluetooth enabled, interacting with another Bluetooth enabled device) • Cannot distinguish proximity with high vs low risk of transmission
**Opt-in application data:**				
• Applications using Bluetooth and/or GPS location data to track interactions between individuals collect data passively through enabled phones and/or actively when users respond to prompts • Application specific, but could consist of time stamp, distance, duration of interaction, questionnaire responses	• Proximity Network (identified contact chains)	• Contact tracing to facilitate quarantine of potentially infected persons	• Enable rapid tracing and quarantining of exposed individuals with fewer resources • Allow for measured behavior to be linked to an individual’s infection status	• Low tolerance for missing data; unclear ability to sufficiently scale up • Cannot distinguish proximity with high vs low risk of transmission • Selection bias in the population for whom data is available (smartphone users, possibly Bluetooth enabled, opted into and compliant with application, interacting with another user opted into and compliant with application)

### Evaluating current interventions and monitoring their release

The most widely used application of mobile phone data in public health to date is the use of telecom geolocation data to track population movements^11,12^. Mobile phone operators routinely collect Call Detail Records (CDRs) that contain a timestamp and GPS location with a unique identifier for all subscribers. These data thus are typically readily available and offer high coverage to estimate mobility patterns of individuals using their mobile devices. We note that similar time-resolved GPS location data may be passively collected through certain applications, though typically for only a subset of subscribers that may introduce further bias.

CDRs can be used to generate a number of metrics for characterizing large, population-level mobility patterns. Origin-Destination (OD) matrices reflect the number of times a trip is made between two locations (of varying spatial resolution) in a certain period. These matrices can be analyzed over time to detect temporal trends (i.e., holidays, seasonality, weekday vs weekend) and regular hotspots of attraction. These spatial and temporal flows of individuals between locations, including the magnitude and frequency of these movements, can be used to understand the risk of importation from areas with ongoing outbreaks to areas without sustained transmission where there is a risk of reintroduction and resurgence. Aggregate flows can also be used to retrace the likely introduction and spread of an outbreak in new areas and to inform future projections of disease risk or burden across space and decision making around the design and implementation of travel restrictions or increased surveillance.

Aggregate mobility patterns may also be critical pieces of evidence when evaluating the effectiveness of various NPIs. Most NPIs are reliant on modifying physical behavior. Monitoring the volume, frequency, and average distance of flow during interventions can be used to directly quantify the adoption and effect of these interventions, and identify areas of high potential risk to target with different interventions. There are already identified associations between reductions in population-level mobility within and between different locations and COVID-19 incidence^6,10,29^, though further exploration of which population-level metrics are most closely related to changes in disease risk and whether these associations are sustained throughout an outbreak is needed^30^. These associations would ideally be interrogated to identify individual behaviors associated with mobility measures that are also associated with individual risk of COVID-19.

The effect on NPIs can also be monitored through subscriber density metrics that combine the recorded GPS location and timestamp of CDRs to capture the real-time population density and identify potential hotspots. When using finer-scale GPS location data, these density metrics may quantify the likelihood or frequency that users came into proximal contact. A third metric derived from CDR or GPS location data, the radius of gyration, quantifies the range over which a single person may travel in a specified time period. Importantly, the data required for these applications are non-identifiable; they cannot be used to identify any given individual’s interactions, but provide population-level insight into the average clustering and movement of individuals. These metrics, along with traditional OD matrix flows, were recently employed in Italy as a way to evaluate the impact of its national lockdown^31^. Traffic flow between provinces and probability of colocation were reduced initially in the northern provinces, where the COVID-19 outbreak was first observed, a clear signal of reactive social distancing. As the epidemic progressed, and especially once the national lockdown was enforced, the entire country saw a reduction in traffic between provinces; however, the probability of colocation remained highly dependent on province and was likely attributed to the number of cases reported in each province. Interestingly, the average distance traveled by individuals was significantly reduced across all provinces after the initial outbreak was confirmed.

The use of Bluetooth data (records of proximal interactions between Bluetooth-enabled devices) to quantify physical clustering or real-time density of subscribers at small spatial scales (e.g., zip codes) and fine temporal resolution has been explored for the purposes of contact tracing (see below). The use of these data has been considered less for population-level analyses, though it offers another source of information on behavioral changes under different NPIs. When activated, mobile phones will emit a Bluetooth beacon that is detected by other activated phones. When two Bluetooth-enabled devices are within range, the date, time, distance and duration of interaction can be recorded. The frequency or number of these interactions (analyzed anonymously to form, broadly, measures of clustering or proximal interaction rates over time) may be important given the role of sustained interaction or overcrowding of individuals^32–34^ and contact structure in SARS-CoV-2 transmission^35^. Furthermore, Bluetooth data in combination with GPS data or a network of Bluetooth sensors can be used to quantify the amount of time people spend at home or other identified locations when lockdown measures are in place to determine if policies are effective.

These data and measures of population-level mobility or clustering patterns would be exceedingly difficult to collect on a similar scale without mobile phone data. These data are often continuously collected, in near real-time, allowing for continued analysis as an outbreak unfolds. Importantly, though, a baseline understanding of contact or clustering patterns prior to any interventions is necessary to inform estimates of intervention impact.

### Facilitating contact tracing

Opt-in applications (apps)^36–42^ that rely on digital approaches to enumerate and contact individuals who may have been in proximity with someone infected with COVID-19 have been proposed to increase efficiency and decrease the very large burden of manual contact tracing programs^43–45^. By enabling rapid tracing of perhaps higher proportions of affected individuals, these apps can reduce the amount of time that a potentially infected person would have to infect others, particularly in asymptomatic or pre-symptomatic phases of infection^46^. Most contact tracing apps collect Bluetooth and/or GPS location data to create trails of contacts over a moving time window (14-28 days). Unlike the data needed to understand population-level, aggregated behaviors described above, these data must be linked to single individuals and capture pairwise interactions with other identifiable individuals. Once a case has been identified, they are added to a list of infected users that is queried by the other phones in the network. If the infected user is detected in the trail of contacts, then the user and their contacts are alerted, either by the app or by a public health official, to initiate isolation and quarantine.

This contact tracing process occurs either in a centralized manner, where user information is sent to a remote computer where matching occurs, or in a decentralized manner, where the matching process occurs on the user’s phone. In order for these approaches to feed directly into public health decision making, a direct line between the developers, public health response teams, and users needs to be put in place. This will also be key to mitigating any privacy concerns, which should be dealt with in a transparent and direct manner. Although there has been little discussion to date, routinely collected, individually-identifiable Bluetooth or fine-scale GPS location data may also be used to infer and quantify high-resolution proximity network structures which may further inform contact tracing efforts, but will also raise additional privacy concerns^47,48^.

### Frameworks to process and analyze mobile phone data

Luckily, computing resources and methods to analyze and extract these data will not likely be the limiting factor in these instances. Groups such as Flowminder and Telenor Research Group have worked for multiple years to develop more streamlined processes to analyze these data, particularly aggregate mobility data, that are able to directly interface with mobile phone operators. Flowminder has produced a suite of CDR aggregates, such as counts of active subscribers per region or counts of travelers, that can then be used to calculate indicators of mobility, such as crowdedness, population mixing, locations of interest, and intra-/inter-regional travel^49^. The code to extract these metrics is publicly available at^50^. Telenor Research Group works directly with mobile phone operators to provide researchers with spatially aggregated CDR/mobility data^51^. Facebook’s Data For Good program provides aggregated mobility data to researchers that come from their subscribers, and companies like Cuebiq provided mobility data for a number of COVID-19 studies that summarize the distance users travel or the proportion of users that stay at home^52^. These existing frameworks - not only the analyses, but also the privacy considerations and data sharing agreements - will provide standardized methods that facilitate integrating mobility data into intervention assessments.

### Data privacy

Various forms of identifiable personal information are generated when using mobile phones, including names, identification numbers, fine spatial and temporal data on where the device was used, other users’ identification numbers who may have been detected by Bluetooth, and personal details that might be entered into an app. In light of the growing number of digital privacy concerns and regulations, one must carefully consider the exact form and use of mobile phone data being collected against the legal and ethical need to protect users’ data security and confidentiality. While maintaining user confidentiality is often seen as a hindrance to the use of mobile phone data, in that it limits the use of individual-level data and typically requires aggregation to coarse spatial and temporal resolutions, there are a number of existing frameworks that can help provide guidance for the effective, privacy-conscious use of mobile phone data^53^.

Exactly which model of data privacy will best suit the use of mobile phone data for COVID-19 response will depend on the exact form and proposed use of the data. As discussed above, there already exist many data processing and analysis frameworks to provide anonymized indicators of population mobility. These standard procedures, though, could result in aggregated data with insufficient spatial and temporal resolution to be effective for monitoring the spread of SARS-CoV-2. Privacy regulations, such as the European Union’s General Data Protection Regulation (GDPR)^54^, offer exceptions for the use of non-anonymous data that may be needed for other response efforts. For example, opt-in applications for contact tracing may seek consent of the data subject to collect and analyze identifiable data, though the ability to scale opt-in approaches to a wide enough population and to maintain user compliance and participation remains unclear. GDPR and other regulations also provide an exception for anonymization of data to be used in public service, but the regulatory hurdles to gain this exception can be substantial and would require clear use policies and applications for these data. The use of mobile phone data, particularly forms such as those proposed through contact tracing applications, must be weighed against the possible infringements of privacy and civil liberties versus the potential public health benefit.

## Capturing epidemiologically-relevant behaviors with mobile phone data

Both the ability to capture behavioral patterns in a large proportion of the population and the potential scalability of these approaches are some of the most promising aspects of mobile phone data^11,12^. The potential for broad expansion in the collection and availability of mobile phone data requires an understanding of exactly which behaviors are captured and whether these behaviors are valid measurements of interactions relevant for infectious disease transmission (Table 2). The validity of these behavioral metrics needs to be evaluated in the specific context of their application, including the spatial and temporal scales of the data and the proposed public health actions or policies informed by these data.

**Table 2:** Epidemiologically-relevant behaviors captured in mobile phone data.

	What is captured?	What is not captured?
Spatially and temporally aggregated mobility (CDRs, GPS)	• Changes in population-level mobility and clustering behaviors in response to NPIs • Rates at which individuals move between locations • Potential transmission links between locations • Hourly or daily movements	• Changes in individual behavior, trajectories, or specific routes • Differences in how individuals use their phone • Distinction between movement with high vs low risk of transmission • Transmission chains within locations
Proximity networks (Bluetooth, contact tracing applications)	• Relationship between individual’s behavior and infection status • Fine-scale clustering and contact data	• Distinction between proximate individuals who are in direct contact or not in contact • Non-proximal interactions that may be involved in transmission

Many natural experiments are now occurring as various NPIs are implemented and lifted, which could be systematically and passively measured via mobile phone data to guide decision makers monitoring the effectiveness and implementation of various NPIs in real time. CDR data may offer one of the best assessments of changes to population-level mobility and clustering behaviors in response to NPIs at potentially fine spatial and temporal scales. These data, though, are only relevant to disease transmission if we can assume that these aggregate behaviors capture the movement of infected, and potentially infectious, individuals. Individual behavior may change in response to real or perceived illness in ways that are not easily captured in aggregate metrics. These aggregate measures are also unable to distinguish movement with high risk of transmission (e.g., shared public transit without appropriate protective equipment) from movement with low risk (e.g., travel by personal vehicle with appropriate social distancing), and therefore will not fully capture the spectrum of behavioral changes that may reduce disease risk.

All forms of mobile phone-based tracking are only able to capture proxies of movement, in that they track a device rather than an individual. Compared to other measures of human mobility (surveys, direct observation), mobile phone data tend to more completely capture the movement of individuals within the study population. However, differences in how individuals use their phone may introduce important biases, particularly when attempting to assess changes in behavior across time or across populations. For example, mobile phone data may be unable to capture an increasing proportion of individuals staying at home or at work following restrictions on non-essential travel, where they may be more likely to use Internet-based communication that does not generate CDRs. Similarly, there may be differences in how individuals of different ages or in different regions use their phones, which will affect the validity of mobile phone metrics in these populations. Mobile phone data cannot distinguish between multiple people using a single phone or SIM card (either of which may be used as a unique identifier), nor does it account for users with multiple phones or SIM cards, limiting the ability to make any inferences about the behavior of individuals from CDR data.

The spatial and temporal scales over which these aggregate data are collected also have important implications for their application. Mobility flows derived from CDRs are commonly used in metapopulation transmission models to parameterize the rates at which individuals move between various locations. This application requires that the origin and destination locations be spatial areas within which the exact social contact patterns of individuals can be estimated (e.g., through mass action assumptions or age-specific contact matrices) and which directly relate to relevant public health decisions (e.g., administrative units with a common public health authority). Data availability and privacy regulations often limit the spatial scales on which these locations can be defined, nor is it always clear which locations are most relevant to disease transmission processes. While mobility flows are useful for understanding potential transmission links between these locations, when they can be defined, the spatial aggregation naturally limits their utility in understanding or modeling transmission chains within these locations. Respiratory viruses like SARS-CoV-2 are diseases of close contact; the spatial scale (several meters) over which transmission occurs is many times smaller than what can be explored through aggregate mobility flows (typically aggregated to areas of at least 500 m^2^).

The temporal scale of aggregation is also important. For reasons of privacy and computational efficiency, several hour time steps or daily movements are often calculated, and multi-step journeys over several days are not measured. Similar privacy and efficiency concerns mean that individual trajectories (e.g., moving from A to B to C) are often impossible to measure. As such, aggregate data are used and, though the relative connections between places are typically robust (e.g., flows between A and B), the exact magnitude of travel occurring multiple times per time step or along specific routes (e.g., transit in and out of a capital city) are difficult to capture in aggregate data.

Contact tracing applications require the use of identifiable, fine-scale clustering and contact data to understand the proximal interactions individuals have that may result in disease transmission. The tolerance for missing data in these applications is low; any missed interaction might be a missed transmission event. Unlike aggregated metrics, contact tracing applications allow for measured behavior to be linked to an individual’s infection status, though it is still unclear how to translate the physical proximity of mobile devices to transmission-relevant interactions between individuals. Particularly in dense areas (e.g., apartment buildings), these applications may capture many proximal interactions which have very low risk of transmission, leading to high rates of unnecessary quarantine and associated social and economic costs. It further remains unclear whether SARS-CoV-2 transmission through non-proximal interactions (aerosols, fomites, fecal-oral) plays an important role, compounding the difficulty of defining transmission-relevant interactions that can be captured in mobile phone data.

## Evaluating the ability of mobile phone data to represent populations at risk for COVID-19

A key advantage of mobile phone data is the possibility to quickly collect bespoke data in many areas; particularly in a pandemic, where tailored responses to specific epidemiologic and social contexts are required, use of mobile phone data might require fewer assumptions about the transportability of derived mobility and contact metrics across different populations. Critically, though, data derived from mobile phones only directly captures mobility and contact among those who own and regularly use a mobile device (see Fig. 1b). While there is evidence these measures do reflect movement of individuals without mobile phones on a population level, the exact representativeness of mobile phone data to populations without phones in varied settings for both population-level flows and individual-level clustering patterns remains unclear^55^. Any applications that require smartphone versus general phone use may further limit their generalizability and applicability. Prior work has shown clear sociodemographic and age biases of mobile phone ownership within populations in multiple settings^56,57^. Children and the elderly are frequently under-represented in mobile phone data, and inferences derived from mobile phone users may not be generalizable to these populations. Even in areas where mobile phone ownership is nearly-ubiquitous, data may not be fully representative if collected only from a single operator, which may target a specific population, or from an area with unequal service coverage, which is necessary for CDR and GPS location capture. Issues of service coverage may be particularly problematic in low- and middle-income countries, where there are generally fewer events recorded in CDR data, leading to greater uncertainty and potential for bias in estimates of mobility. Mobile phone owners may not be the primary or sole users of their device, too, further complicating the transportability of these data to particular populations.

Applications with opt-in or opt-out features will have an additional level of selection in the population for whom data is available; this selection could be particularly dangerous if there is clustering in the populations which do not participate in app-based responses or uses that require smartphone ownership. Modeling work has indicated that upwards of 60-80% of individuals must be enrolled in contact-tracing applications to achieve substantial reductions in transmission, but these estimates may be optimistic if chains of transmission are sustained and uninterrupted among clusters of non-participants^46,58^.

There are clear age-specific and social disparities in COVID-19 risk that may dovetail with the potential selection biases in mobile phone data, leading to a critical need to understand where and to what extent these biases may exist. Identifying and quantifying these biases is particularly challenging, though, when there is no clear gold standard against which to validate mobile phone data. Transparent usage and ownership information will allow for exploration and characterization of the populations under study and potential generalizability of results^59^. For example, areas with limited coverage may be excluded from certain analyses, or population weighting may be used to derive appropriate, adjusted estimates of population mobility. Targeted studies to understand how aggregate mobility or contact patterns differ by important demographic groups (e.g., the elderly, essential care workers) may be needed to capture the most epidemiologically-relevant and high-risk patterns of behavior relevant to COVID-19 response.

## Determining the appropriate resolution for evaluating behavior in a pandemic

Most mobile phone data is used to understand aggregate, population-level behaviors. Though commonly used to describe possible mechanistic drivers of transmission, these aggregate patterns may not be the most relevant to public health responses, particularly if the focus of pandemic response shifts from containment and mitigation to sustained surveillance and perhaps even local elimination that require highly targeted responses. Moreover, it is increasingly clear that the COVID-19 burden is not equally borne throughout the population. Using aggregate mobility flows to estimate population-level reductions in travel will fail to capture increased risk among essential workers unable to stay home. However, there may be promise in aggregating individual-level data (such as contact tracing data) that may provide additional epidemiologically relevant information.

The COVID-19 pandemic has therefore led to an increased push to utilize mobile phone data to quantify fine-scale, individual-level contact and clustering patterns. These kinds of data have historically been key features of outbreak control, but have involved labor-intensive, manual contact tracing, surveillance, and outbreak investigations. The ability to standardize and streamline the collection, analysis, and application of individual-level data for outbreak response could reduce the current burden on public health officials and is being actively explored by multiple teams.

Developing this ability is not without its challenges, though. Collecting potentially sensitive identifiable data, perhaps passively or without opt-in consent, requires a deep, careful understanding of the legal, ethical, and privacy concerns surrounding the collection and use of these data relative to the potential public health applications and benefits. In all cases, transparent data policies must be used to ensure community engagement and appropriate use and dissemination of collected data. Opt-in features may be necessary to adequately maintain privacy and respect for persons, but will likely impact adoption of data collection and may result in severe selection biases.

In fact, the pitfalls of non-representative study populations and demographic biases may become more pronounced with individual data at finer spatial and temporal scales than aggregate population-level mobility patterns. For opt-in approaches, complementary research into who adopts, uses, and complies with platforms collecting and using individual-level data will be key to assessing their effectiveness. There must additionally be strategies to ensure engagement and representation of high-risk groups in the design and roll-out of these platforms to mitigate the most severe consequences of the pandemic.

## Integrating mobile phone data into decision making

Although mobile phone data show great promise for characterizing population- and individual-level behaviors, there remains considerable uncertainty around how to appropriately account for the patterns and potential biases within these data. Policy decisions informed by mobile phone data must consider which populations and which behaviors are or would be excluded from data and which approaches in the design, collection, analysis, or interpretation of mobile phone data can help confront these biases. Care must also be taken to collect and analyze mobile phone data at the appropriate scale for the decision at hand; mobile phone data can potentially provide highly detailed data at various levels of spatiotemporal resolution and aggregation, and the exact data and inferences required will change with the specific decisions and challenges being confronted.

Many important, practical questions must also be addressed before widespread use of mobile phone data is possible, including: How will the data be used? Who will have access to these data? How long will the data be used and stored? Developing solutions that maintain the privacy of subscribers and follow appropriate regulations while protecting data sovereignty for operators and ensuring the most complete, informative data is made available to researchers and stakeholders will be key to the widespread use and adoption of mobile phone data. Collaborative groups such as the COVID-19 Mobility Network and European Commission are avenues that can standardize and expedite the dissemination and use of mobile phone data for public health response^60^. However, careful, context-specific application of standard methods is necessary to translate them into policy, especially when considering regulatory differences and changing public health priorities across countries. This points to a broader challenge in the use of mobile phone data to explore mobility and contact patterns across international borders. These data typically only include information on movement or clustering within a given country, but cross-border travel will likely be an increasingly important consideration in COVID-19 response as travel restrictions are eased.

Effective use of mobile phone data will require direct, iterative collaboration between mobile phone operators, researchers, and public health officials. This form of reciprocal collaboration will facilitate rapid processing and bespoke analysis of these data; this can also help ensure the continued participation of mobile phone operators, who typically participate in such public health initiatives as a form of social responsibility. The near real-time nature of mobile phone data is a key feature that must be appropriately leveraged to inform response in rapidly-changing situations. At the same time, it is necessary to contextualize the analysis and interpretation of these data as they are integrated into public health decision making. Without appropriate communication, mobile phone data at best will be of limited use and at worst could lead to misguided decisions informed by bad data.

It must be acknowledged, though, that while mobile phone data contain challenges, the perfect data are rarely available in emergency situations. An informed response must consider all available evidence, of which mobile phone data can be a key component at every stage. Mobile phone data are not a panacea, but they may serve as an important complementary approach that can facilitate rapid, early assessments of intervention impacts along with existing surveillance systems tracking disease burden. Mobile phone apps can serve as an important technological resource in contact tracing efforts, and continued monitoring of clustering and contact patterns through mobile phone data will complement active surveillance efforts as the current pandemic wanes.
